# Assessment of Density Functional Approaches for Carbon Dioxide Dimerization and Solid-State Properties

**DOI:** 10.1021/acsomega.6c00095

**Published:** 2026-03-17

**Authors:** Elizane E. de Moraes

**Affiliations:** Instituto de Física, Universidade Federal da Bahia, Campus Universitário de Ondina, Salvador 40210-340, Bahia, Brazil

## Abstract

We investigated the performance of various exchange–correlation functionals implemented in SIESTA, including van der Waals (vdW) dispersion corrections, specifically the (i) BH, (ii) C09, (iii) KBM, (iv) LMKLL, and (v) VV variants, as well as the generalized gradient approximation (GGA), to describe the interactions of CO_2_ homodimers using density functional theory (DFT). To the best of our knowledge, the application of these functionals to CO_2_ systems has not been previously reported in the literature. Accordingly, we calculated the interaction energies of the CO_2_ homodimers, their vibrational modes, and the solid-phase properties using vdW-DF3-type functionals. Among the dimer configurations analyzed, the T-shaped (TS) and parallel-displaced (PD) geometries were identified as the most stable. Notably, the qualitative and quantitative behavior of the vdW-DF-C09 functional for the PD configuration showed excellent agreement with results obtained from high-level CCSD­(T)/SAPT calculations. Furthermore, vdW-DF-C09 successfully reproduced experimental data for the solid phase of CO_2_, including its symmetric, asymmetric, and bending vibrational modes. A comparative analysis of different van der Waals (vdW) functionals offers valuable insights for parametrizing force fields suitable for classical simulations. These findings provide a reliable benchmark for CO_2_–CO_2_ interactions and may serve as a reference for future force-field development and multiscale modeling studies.

## Graphical abstract

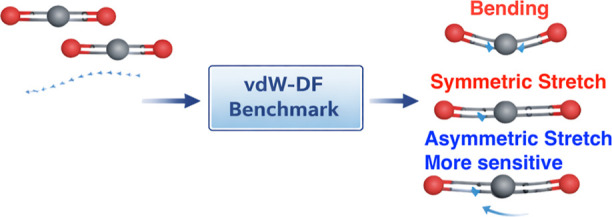

## Introduction

Carbon dioxide has garnered extensive scientific interest in various fields.^1–9^ Restrictions on GHG emissions are driving the need for environmentally friendly and innovative industrial processes for gas separation. Within the Carbon Capture and Storage (CCS) challenge, gas separation in membranes has been addressed by several studies.^10–18^

A fundamental challenge is the enormous number of materials that can be considered for gas separation processes. Computational simulations can be used to predict physicochemical properties, thus making the design process less expensive and more reliable. However, the development of accurate force fields remains a limiting factor for many applications. In this context, it is crucial to understand the intramolecular and intermolecular interactions that govern the CO_2_ behavior across different phases. In particular, weakly bound systems such as CO_2_ dimers, which are dominated by quadrupole–quadrupole interactions and dispersion (induced dipole) effects, pose a significant challenge for theoretical methods. Although these interactions are weak, they play a fundamental role in determining the structure and energetics of clusters and condensed phases.

The carbon dioxide dimer has been the object of several experimental^19–23^ and theoretical studies.^24–29^ Experimentally, the carbon dioxide dimer has been the subject of controversy for many years in the debate over its most stable configuration using infrared, Raman, and inert gas matrix spectroscopy techniques. Many experimental studies have identified the T-shaped (TS, C2v) structure as the most stable. Later on, Jucks et al.^19,20^ finished by showing that the equilibrium structure is a parallel displacement (PD, *C*_2*h*_ symmetry) and that the T-shaped structure is a transition state. Additionally, configurations TS and PD were observed in both the liquid and gas phases of the carbon dioxide dimers. Then, other configurations were identified through the contribution of computer simulations in the liquid phase.^29^ In addition, electron diffraction studies^30^ of larger clusters in the range of 102–105 CO_2_ molecules per cluster (CO_2_)­n revealed the crystal Pa3 structure dictated by the arrangement of quadrupolar molecules on a face-centered cubic lattice.

Theoretical descriptions of the intermolecular interaction, describing the forces of CO_2_ molecules, have a long history because of the fundamental nature and practical importance of this system. Among more recent ab initio works, Kalugina et al.^29^ performed a comprehensive study using high-level CCSD­(T)/aug-cc-pVXZ (X = D, T, Q, CBS) theory for the CO_2_ dimer, where they emphasize the importance of (i) the potential energy surface (PES) of the PD and side-by-side (SS) configurations and (ii) using large basis sets and the van der Waals (vdW) interactions. In addition, Tsuzuki et al.^24^ and Bock et al.^27^ in the MP2 level in small to moderate basis sets, and Bukowski et al. employed SAPT combined with aug-cc-pVXZ basis sets (X = D, T, Q, CBS).^25^ Using SAPT (symmetry-adapted perturbation theory), the importance of correlation energy and basis-set superposition error (BSSE) in energy calculations for dimers was highlighted. In these works, PD and TS configurations were identified as being the most stable among the CO_2_ dimers. However, these works do not show the PES profile for other CO_2_ dimer configurations.

Investigating the interactions of carbon dioxide homodimers can lead to an improved understanding of its behavior in a large number of applications, such as (i) industrial solvent; for instance, CO_2_ can be an inexpensive and environmentally harmless solvent for supercritical fluid extraction; (ii) parametrization of force fields suitable for classical simulations; and (iii) optimizing the properties of materials designed for CO_2_ capture and storage.

In the present work, ab initio calculations were used to perform a benchmark of the exchange and correlation functionals that describe the CO_2_ intermolecular vdW interactions. In this work, a benchmark study of several exchange–correlation functionals was performed for two complementary reasons. First, we identify exchange–correlation functionals capable of accurately describing minimum-energy configurations of CO_2_ within density functional theory (DFT) and compare the results with more sophisticated theoretical approaches. Second, since the focus is on intermolecular interactions, the interaction energy must correctly approach zero at large intermolecular separations. This requirement is essential for a consistent and physically meaningful description of the CO_2_–CO_2_ interaction energy.

## Methodology and Computational Details

Our calculations were performed within the framework of density functional theory (DFT)^31^ as implemented in the SIESTA program.^32^ We have used Troullier–Martins norm-conserving relativistic pseudopotentials in the Kleinman–Bylander nonlocal form.^33,34^ The investigated functionals were the van der Waals (vdW) dispersion correction with the (i) BH,^35^ (ii) C09,^36^ (iii) KBM,^37^ (iv) LMKLL,^38^ and (v) VV^39^ and the generalized gradient approximation (GGA) with the Perdew, Burke, and Ernzerhof (PBE).^40^ For more information, see Supporting Information. To represent the charge in the real space, a grid cutoff of 300 Ry was used. The structures were relaxed until the residual forces were less than 0.05 eV/Å.

For the interaction energy *E*_int_, calculations are given by the following equation:

1 $$E=E_{CO_{2}-CO_{2}}-2E_{CO_{2}}$$

where the first term stands for the total energy of the system in a given confirmation and the second term represents the ground state energy of 2 separate CO_2_ molecules. The values taken as a reference for this work, when the binding energy is negative, mean that the system is attractive.

In the second part of this work, the aim is to benchmark the functionals for describing the vibrational modes of CO_2_ and the bulk modulus of the cubic phase. The vibrational modes calculation was carried out using the PHONOPY package^41^ with an atomic displacement of 0.001 Bohr. The forces due to the displacement are calculated within the SIESTA code, and then PHONOPY^41^ is used to extract the force constants necessary to obtain the dynamical matrices.

The bulk modulus for the cubic phase Pa3 (205) was determined by fitting the total energy versus volume using the Murnaghan^42^ equation of state for the cubic phase and using *k*-point samplings of 11 × 11 × 11. At the selected volume, the considered phase was fully relaxed to its equilibrium configuration by calculating atomic forces. Subsequently, the equilibrium structural properties were evaluated by fitting the total energy versus volume using the Murnaghan^42^ equation of state.

## Results and Discussion

First, representative parallel and T-shaped intermolecular configurations for carbon dioxide homodimers were evaluated (Figure 1). Thereafter, the potential–energy curves of these configurations were calculated as functions of the distance *r* between the homodimers.

**Figure 1:**
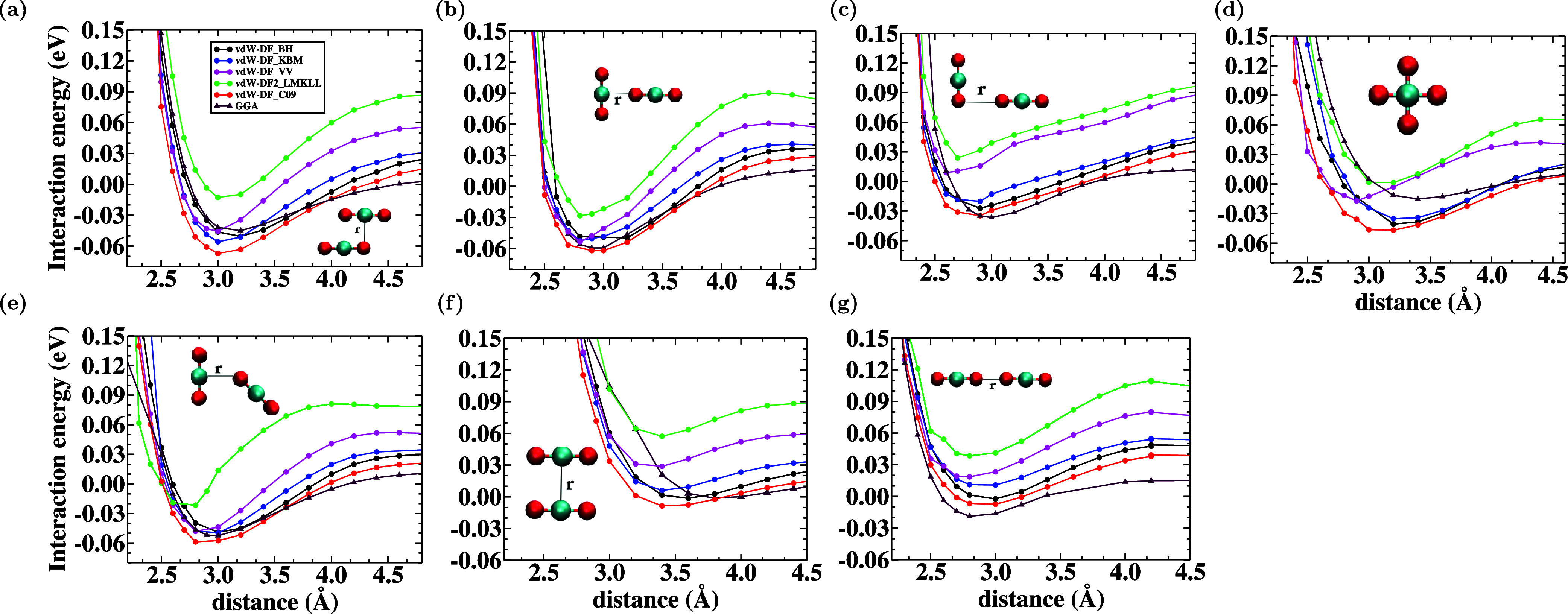
Structural configurations and interaction energy curves for the carbon dioxide homodimers: (a) parallel displacement (PD), (b) T-shaped (TS), (c) T-shaped displaced one (TSD_1_), (d) cross (CR), (e) T-shaped displaced two (TSD_2_), (f) face to face (FF), and (g) side by side (SS) computed with different functionals. The black, blue, magenta, green, and red circles and indigo triangle symbols represent data obtained from vdW-BH, vdW-KBM, vdW-VV, vdW-LMKLL, vdW-C09, and GGA functionals, respectively.

For the CO_2_–CO_2_ interaction, seven basic configurations were considered: (a) parallel displacement (PD), (b) T-shaped (TS), (c) T-shaped displaced one (TSD_1_), (d) cross (CR), (e) T-shaped displaced two (TSD_2_), (f) face to face (FF), and (g) side by side (SS). The interaction energy curves for the carbon dioxide homodimers are shown in Figure 1.

The side-by-side configuration shown in Figure 1g is the least attractive since its major energy configuration is the repulsion of the O–O repulsion. The second-order Møller–Plesset (MP2) theory fails to capture the small attractive part of this configuration,^24^ while theories studied in this work, such as DFT-vdW-BH, DFT-vdW-C09, and GGA, and also CCSD­(T)/aug-cc-pVXZ (X = D,T,Q,CBS)^29^/SAPT^25^ capture the small attractive behavior of this arrangement, around −0.0023, −0.0074, −0.019, – 0.0034,^29^ and −0.0062 ^25^ eV, respectively.

The parallel displaced and T-shaped configurations illustrated in Figure 1a,b show the lowest energies when compared with other configurations, around −0.067 eV at 3 Å and −0.061 eV at 2.8 Å for vdW-C09, respectively. The calculations show that the interaction energy surface is very shallow. The attractive behavior at very low distances in this case can be explained in terms of the favorable C–O electrostatic interaction and not the repulsion of O–O quadrupoles in these arrangements. The different functional vdW types and GGA correctly describe the interaction energy, with the exception of the vdW-LMKLL, which underestimates the CO_2_–CO_2_ binding energy. The qualitative and quantitative behavior of the functional vdW-C09 for the PD configuration is in agreement with the works by Kalugina et al.^29^ and Bukowski et al.,^25^ who used the high-level CCSD­(T)/aug-cc-pVXZ (X = D,T,Q,CBS)/SAPT theory.

Among the TSD_1_, cross, and TSD_2_ configurations shown in Figure 1c, 1d, and 1e, respectively. The third is more stable with an interaction energy value of vdW-C09 of −0.05876 eV at 2.8 Å. Due to the favoring of C–O interactions in this arrangement, for these configurations, the different functional vdW types and GGA correctly describe the interaction energy, with the exception of vdW-LMKLL and vdW-VV, which underestimate the CO_2_–CO_2_ binding energy and distance. The difference between the quantitative behavior of these curves is attributed to the way the functional vdW-type was parametrized. The face-to-face configuration, as shown in Figure 1f, exhibits repulsion between two oxygen dipoles, resulting in a low and attractive binding energy. The functionals that can capture the interaction energy correctly are vdW-BH, vdW-C09, and GGA, with values of around −0.0013, −0.0085, and −0.00128 eV, respectively. This small, attractive behavior is observed in the work of Bock et al.^27^-CCSD­(T) (around −0.0028 eV), while the MP2 theory fails to capture the small attractive part of this configuration.^24^

Table 1 shows the values of the interaction energy between the CO_2_ homodimers for the seven configurations with different functionals. When compared with more sophisticated theories, for instance, high-level quantum calculations such as MP2, SAPT, CCSD­(T), and vdW-DF methods,^26–29^ the energies computed by the vdW-C09 functional are able to capture the qualitative and quantitative behavior of these sophisticated theories for calculating the binding energy between CO_2_ homodimers. In this work, TS and PD are the preferred configurations. This behavior is also observed in the studies.^26–29^ We hope our comparison between the functional van der Waals types can offer insights into the studies of industrial solvents, the parametrization of force fields suitable for classical simulations, and optimizing the properties of materials designed for CO_2_ capture and storage.

**Table 1:** Exchange–Correlation Functional, Configurations, Interaction Energies, and Distance for Carbon Dioxide Homodimers

configurations	distance (Å)	energy (eV)
Parallel Displaced		
vdW-BH	3.2	–0.050
vdW-KBM	3.0	–0.055
vdW-VV	3.0	–0.045
vdW-LMKLL	3.0	–0.012
vdW-C09	3.0	–0.067
GGA	3.2	–0.045
T-Shaped		
vdW-BH	2.9	–0.049
vdW-KBM	3.0	–0.052
vdW-VV	2.8	–0.053
vdW-LMKLL	2.8	–0.027
vdW-C09	2.8	–0.061
GGA	2.9	–0.058
T-Shaped Displaced One		
vdW-BH	3.0	–0.024
vdW-KBM	2.9	–0.020
vdW-VV	2.6	0.008
vdW-LMKLL	2.8	–0.009
vdW-C09	2.9	–0.034
GGA	3	–0.036
Cross		
vdW-BH	3.2	–0.040
vdW-KBM	3.2	–0.035
vdW-VV	2.9	–0.017
vdW-LMKLL	3.2	0.001
vdW-C09	3.2	–0.046
GGA	3.2	–0.011
T-Shaped Displaced Two		
vdW-BH	3.0	–0.049
vdW-KBM	2.8	–0.047
vdW-VV	2.8	–0.048
vdW-LMKLL	2.8	–0.021
vdW-C09	2.8	–0.058
GGA	3.0	–0.052
Face to Face		
vdW-BH	3.0	–0.002
vdW-KBM	3.0	0.010
vdW-VV	2.8	0.018
vdW-LMKLL	2.8	0.038
vdW-C09	3.0	–0.007
GGA	2.8	–0.018
Side by Side		
vdW-BH	3.6	–0.001
vdW-KBM	3.4	0.006
vdW-VV	3.4	0.028
vdW-LMKLL	3.4	0.057
vdW-C09	3.4	–0.008
GGA	3.8	–0.001

Later on, the vdW types and GGA functionals were employed for calculations of the vibrational energy levels of the molecule of CO_2_. The fundamental frequencies of the intramolecular modes are expected to be near those of their CO_2_ progenitor, for which the frequencies of the asymmetric stretch, symmetric stretch, and bend are 2349, 1332, and 667 cm^–1^,^29,43^ respectively, as shown in Figure S1 (see Supporting Information). Analyzing the bending vibrational and symmetric stretch frequencies, the functionals that best describe these frequencies are the vdW-BH in black and vdW-C09 in red. In the case of asymmetric stretching, the functional that best describes this frequency is vdW-KBM in blue. In the case of the vdW-C09 functional, the bend and symmetrical peaks are in agreement with the experiment around 1332 and 667 cm^–1^, respectively. However, the frequency for the asymmetric stretch is underpredicted by approximately 25 cm^–1^.

In this study, different exchange–correlation functionals were analyzed for the cubic phase Pa3 (205).^44^ This phase was chosen because, in the literature, it is the most stable phase for dry ice,^45^ and it still requires evaluation with van der Waals interactions using other exchange–correlation functionals. The equilibrium lattice parameters (*a*, *b*, and *c*), bulk modulus, and their derivatives were determined by fitting the computed total energy versus volume corresponding to each exchange–correlation functional. Figure S2 shows the fitted curves (energy as a function of volume) for the cubic phase (Supporting Information). Also, the relative structural parameters of each calculated exchange–correlation functional were listed in Table 2. All the exchange–correlation functionals produced the lattice parameters of the solid CO_2_ cubic phase above 1–1.5% of the experimental value.

**Table 2:** Calculated Values of Volume, Energy, Bulk Modulus, Pressure Derivatives (*B*′), and Lattice Parameters of the Nonmolecular Solid CO_2_ Compound Using vdW-BH, vdW-KBM, vdW-VV, vdW-LMKLL, vdW-C09, and GGA Functionals Given in the Cubic Phase^a^

XC functional	*V*_0_ [Ang^3^]	*B*(V_0_)[GPa]	lattice
BH	167.7331	7.9354	5.5149
KBM	167.1023	7.6194	5.508
VV	169.5918	6.5915	5.5352
LMKLL	168.0791	7.4023	5.5187
C09	167.4869	7.7345	5.5122
GGA	167.9168	7.8225	5.5169
Exp	165.85		5.4942

a Compared to other experimental data.^44^

## Conclusions

This article presents a study of the carbon dioxide dimer motivated by the need to model processes occurring in gas separation. In particular, this study highlighted the importance of van der Waals interactions for carbon dioxide by accurately describing the exchange–correlation functional, as verified through a benchmark using density functional theory. We discuss as an absolute novelty the energy interaction between the CO_2_ homodimers, the vibrational modes, and the solid phase using vdW-type functionals: (i) BH, (ii) C09, (iii) KBM, (iv) LMKLL, and (v) VV version, and the generalized gradient approximation version. In the calculation of the interaction energy between the dimers, we found that the T-shaped and displaced parallel configurations are the most stable. The attractive behavior at very low distances, in this case, can be explained in terms of the favorable C–O electrostatic interaction and not the repulsion of O–O quadrupoles in these arrangements. In this context, functional vdW-C09 was able to describe the qualitative and quantitative behavior of the interaction energy between the studied configurations.

For vibrational modes, the bending and symmetric stretch frequencies, the functionals that best describe these frequencies, vdW-BH and vdW-C09, agree with experiments at around 1332 and 667 cm^–1^, respectively. In the case of asymmetric stretching, the functional that best describes this frequency is vdW-KBM. However, the frequency for the asymmetric stretch is underpredicted by approximately 25 cm^–1^. For the study of the cubic phase of dry ice, the functionals employed in this work were able to describe the quantitative behavior of energy versus volume correctly, and the lattice parameters were above 1–1.5% of the experimental value. These results provide a consistent benchmark for CO_2_–CO_2_ interactions and offer a reference for future force-field development and multiscale modeling studies.

## Supplementary Material



## Notes

Vibrational frequencies, additional information about the cubic phase structure for all functionals considered in this study, and details regarding the van der Waals density functional used in this study (PDF)
