# Supplementary material for: Assessment of Density Functional Approaches for Carbon Dioxide Dimerization and Solid-State Properties
Source: ACS Omega. 2026 Mar 17;11(12):19752–7. doi: 10.1021/acsomega.6c00095 (PMC13044839; doi:10.1021/acsomega.6c00095)
Supplement: Supplementary file 1 [file ao6c00095_si_001.pdf]

Supporting Information: Assessment of Density Functional Approaches for Carbon Dioxide Dimerization and Solid-State Properties

Elizane E. de Moraes*

Instituto de Física, Universidade Federal da Bahia, Campus Universitário de Ondina, Salvador 40210-340, BA, Brazil

E-mail: elizane.fisica@gmail.com\\elizanemoraes@ufba.br

Vibrational frequencies and cubic phase

Figure S1 vdW-types and GGA functionals were employed for the calculations of the vibrational energy levels of the molecule of CO_2. Figure S2 shows the fitted curves (energy as a function of

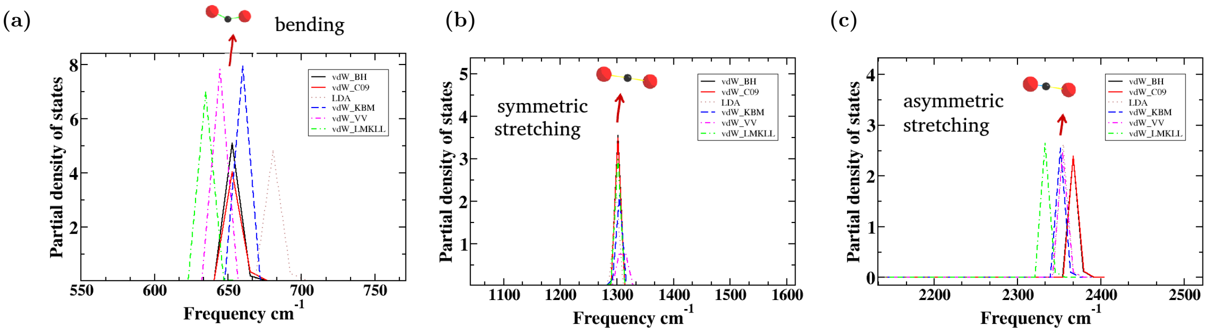

Figure S1: Vibrational frequencies of the CO_2 molecule: (a) bending mode; (b) symmetric stretching; (c) asymmetric stretching, calculated using the vdW-BH, vdW-KBM, vdW-VV, vdW-LMKLL, vdW-C09, and GGA functionals.

volume) for the cubic phase.

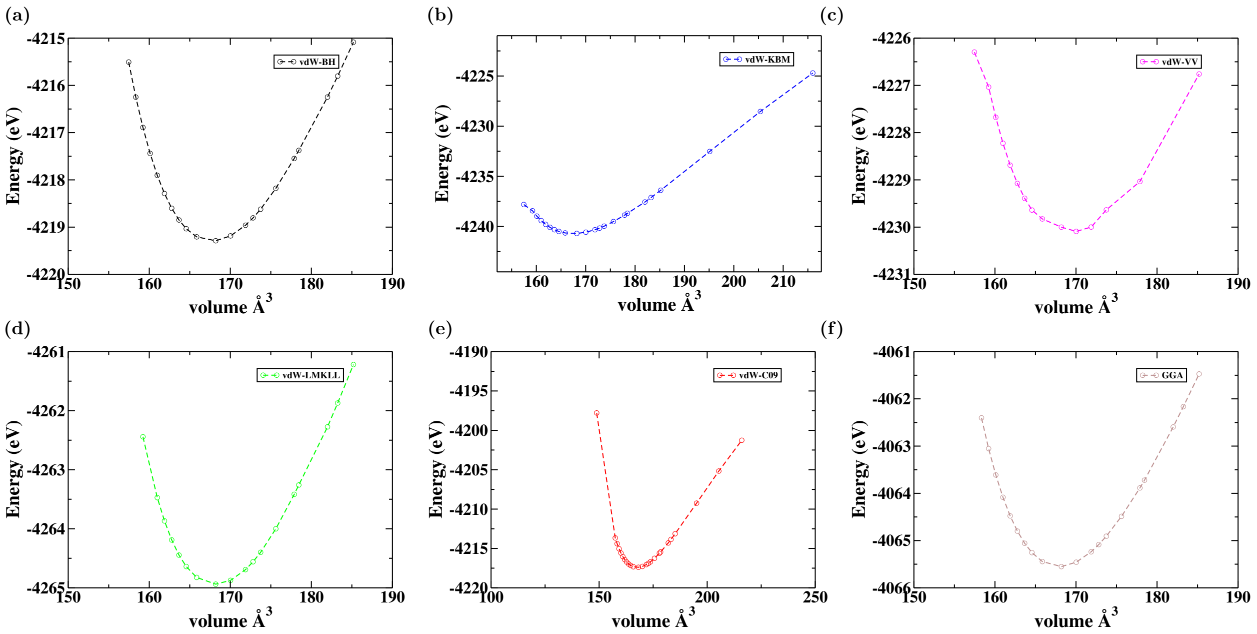

Figure S2: Total energy as a function of volume per formula unit for solid non-molecular CO_2 in the cubic phase (Pa$\bar{3}$, No. 205), calculated using the (a) vdW-BH, (b) vdW-KBM, (c) vdW-VV, (d) vdW-LMKLL, (e) vdW-C09, and (f) GGA exchange–correlation functionals.

van der Waals density functional (vdW-DF)

The DFT frame work, the van der Waals density functional (vdW-DF) is good for both covalent and van der Waals (vdW) interactions in a seamless fashion. In general, vdW-DF exchange-correlation functionals E_{xc}^{vdW} as a sum of exchange energy E_x and correlation energy E_c is expressed as

$$E_{xc}^{vdW} = E_x + E_c = E_x^{GGA} + E_c^{LDA} + E_c^{nl}. \tag{S1}$$

where the correlation contribution is split into LDA and non-local (*nl*) contributions. The exchange GGA functional, is written as

$$E_x^{GGA} = \int drn(r)\varepsilon_x^{unif}(n)F_x(s) \tag{S2}$$

where $\varepsilon_x^{unif}(n) = \frac{-3K_F}{4\pi}$ with $K_F = (3\pi^2(n(r)))^{\frac{1}{3}}$ is the exchange energy for the uniform electron gas and $F_x(s)$ is the enhancement factor r which depends on the reduced density gradient van der Waals exchange-correlation functionals in transition metals. The variable s is a function of the reduced gradient as,

$$s = \frac{1}{2(3\pi^2)^{\frac{1}{3}}} \frac{\bigtriangledown n(r)}{n^{\frac{4}{3}}(r)} \tag{S3}$$

For $s = 0$ the enhancement factor must obviously be 1, since the exchange energy of the uniform gas must be recovered. In the original formulation of the vdW-DF approximation the enhancement factors of PBE and revPBE[1–3] have the same form:

$$F_x^{PBE}(s) = 1 + \kappa - \frac{\kappa}{1 + \frac{\mu s^2}{\kappa}}. \tag{S4}$$

The parameter μ is also the same and so the functionals differ only in the value of the parameter κ. revPBE has a larger value of κ than PBE ($\kappa^{revPBE} = 1.245$, $\kappa^{PBE} = 0.804$), which causes $F_x(s)$ to rise more rapidly with revPBE than PBE. In addition, there are some functionals that $F_x(s)$ that use has the same expression as PBE equationS4, but with values κ and μ between those characteristic of PBE and revPBE, for example optPBE[4] functional.

The gradient expansion approximation is expressed differently for the vdW-C09 exchange functional[5] is given by,

$$F_x^{C09(s)} = 1 + \mu s^2 e^{-\alpha s^2} + \kappa \left(1 - e^{\frac{-\alpha s^2}{2}}\right), \tag{S5}$$

with $\mu = 0.0617$, $\alpha = 0.0483$ and $\kappa = 1.245$. These constants were obtained by a fitting approach to recover the behavior of $F_x(s)$ for small and large values of s. In the $s \to 0$ limit, this functional exhibits the behavior $F_x(s) = 1 + \kappa \left(1 + \frac{\alpha}{2}\right) s^2$, which is quite similar to the behavior presented by PBE and its derivatives.

Another very popular exchange-correlation functional used with vdW-KBM funcional[4] or optB88 functional. This functional is version better than B88[6]the enhancement factor is given

by

$$F_x^{KBM}(s) = 1 + \frac{\mu s^2}{1 + \beta s \arcsin(cs)}, \tag{S6}$$

where $c = 2^{\frac{4}{3}}(3\pi^2)^{\frac{1}{3}}$ the ratio $\frac{\mu}{\beta}$ to lead to increased binding, and μ was changed to 0.22 instead the 0.2743 of B88 functional.[6]

References

(1) Zhang, Y.; Yang, W. Comment on "Generalized gradient approximation made simple". *Physical Review Letters* **1998**, *80*, 890.

(2) Hammer, B.; Hansen, L. B.; Nørskov, J. K. Improved adsorption energetics within density-functional theory using revised Perdew-Burke-Ernzerhof functionals. *Physical review B* **1999**, *59*, 7413.

(3) Kelkkanen, A. K.; Lundqvist, B. I.; Nørskov, J. K. Density functional for van der Waals forces accounts for hydrogen bond in benchmark set of water hexamers. *The Journal of chemical physics* **2009**, *131*, 046102.

(4) Klimeš, J.; Bowler, D. R.; Michaelides, A. Chemical accuracy for the van der Waals density functional. *J. Phys.: Cond. Matter* **2009**, *22*, 022201.

(5) Cooper, V. R. Van der Waals density functional: An appropriate exchange functional. *Phys. Rev. B* **2010**, *81*, 161104.

(6) Becke, A. D. Density-functional exchange-energy approximation with correct asymptotic behavior. *Physical review A* **1988**, *38*, 3098.
